# A Snapshot of the Global Race for Vaccines Targeting SARS-CoV-2 and the COVID-19 Pandemic

**DOI:** 10.3389/fphar.2020.00937

**Published:** 2020-06-19

**Authors:** Colin D. Funk, Craig Laferrière, Ali Ardakani

**Affiliations:** ^1^Department of Biomedical and Molecular Sciences, Queen's University, Kingston, ON, Canada; ^2^Scientific Research Division, Novateur Ventures Inc., Vancouver, BC, Canada

**Keywords:** COVID-19, SARS-CoV-2, vaccine, immune response, coronavirus, clinical trial, public health

## Abstract

A novel coronavirus SARS-CoV-2 causing Coronavirus disease 2019 (COVID-19) has entered the human population and has spread rapidly around the world in the first half of 2020 causing a global pandemic. The virus uses its spike glycoprotein receptor-binding domain to interact with host cell angiotensin-converting enzyme 2 (ACE2) sites to initiate a cascade of events that culminate in severe acute respiratory syndrome in some individuals. In efforts to curtail viral spread, authorities initiated far-reaching lockdowns that have disrupted global economies. The scientific and medical communities are mounting serious efforts to limit this pandemic and subsequent waves of viral spread by developing preventative vaccines and repurposing existing drugs as potential therapies. In this review, we focus on the latest developments in COVID-19 vaccine development, including results of the first Phase I clinical trials and describe a number of the early candidates that are emerging in the field. We seek to provide a balanced coverage of the seven main platforms used in vaccine development that will lead to a desired target product profile for the “ideal” vaccine. Using tales of past vaccine discovery efforts that have taken many years or that have failed, we temper over exuberant enthusiasm with cautious optimism that the global medical community will reach the elusive target to treat COVID-19 and end the pandemic.

## Introduction

Coronavirus disease 2019 (COVID-19) is caused by the novel beta-coronavirus family member coined SARS-CoV-2 (Severe acute respiratory syndrome coronavirus 2) (Oberfeld et al., 2020). In December 2019 (and potentially earlier, though unrecognized), SARS-CoV-2 emerged as a pneumonia-causing disorder in Hubei province, China, most likely the result of natural selection in animal hosts (bats, pangolins) prior to zoonotic transfer (Andersen et al., 2020; Wu et al., 2020a; Zhou P. et al., 2020; Zhu N. et al., 2020). There are now seven members of this viral family known to infect humans with three having the potential to cause severe respiratory disease (Andersen et al., 2020). The two outbreaks preceding SARS-CoV-2 include the first SARS virus emerging in late 2002 in Guangdong province in China (now referred to as SARS-CoV-1), followed by the Middle-East Respiratory Syndrome coronavirus (MERS-CoV) in 2012 in Saudi Arabia (de Wit et al., 2016). SARS-CoV-2 has rampaged exponentially around the world since the start of 2020; the World Health Organization (WHO) declared it a Public Health Emergency of International Concern on January 30, 2020 and a pandemic officially on March 7, 2020 (Sohrabi et al., 2020). At the time of revision of this review (June 7, 2020), infections are at 7 million individuals in at least 195 countries with ≈400,000 in the mortality column^1^. Collateral damage to global economies and many professional business and recreational sectors (travel, hospitality/tourism, sport, etc.) is ongoing and widespread due to governmentally imposed lockdowns.

## SARS-CoV-2

**Figure 1:**
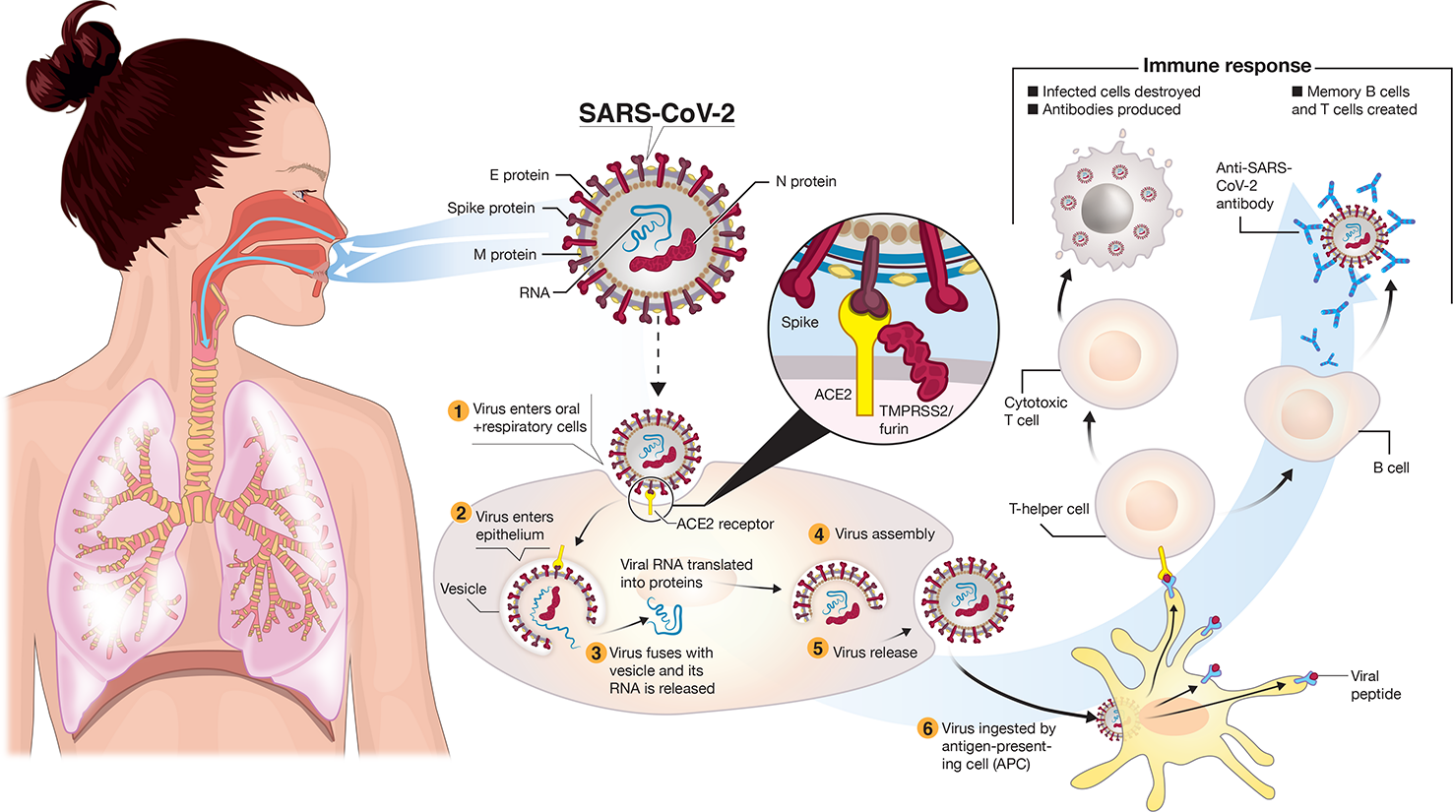
Transmission and life-cycle of SARS-CoV-2 causing COVID-19. SARS-CoV-2 is transmitted *via* respiratory droplets of infected cases to oral and respiratory mucosal cells. The virus, possessing a single-stranded RNA genome wrapped in nucleocapsid (N) protein and three major surface proteins: membrane (M), envelope (E) and Spike, replicates and passes to the lower airways potentially leading to severe pneumonia. The gateway to host cell entry (magnified view) is *via* Spike-converting enzyme 2 (ACE2) interaction with cleavage of Spike in the prefusion state by proteases TMPRSS-2/furin. A simplified depiction of the life cycle of the virus is shown along with potential immune responses elicited.

The virus spreads primarily from respiratory droplets of infected individuals (**Figure 1**) in enclosed spaces, and to a much lesser extent by fomites, to mucosal epithelial cells in the upper airway and oral cavity (Pambuccian, 2020). Here, the virus uses its trimeric Spike protein to latch onto host cell ACE2 (angiotensin-converting enzyme-2) receptor binding sites (**Figure 1**), *via* the receptor-binding domain (RBD) of this glycoprotein in the “prefusion” state (Cyranoski, 2020; Walls et al., 2020; Wrapp et al., 2020; Yuan et al., 2020). Proteases such as TMPRSS-2/furin cleave viral Spike (**Figure 1**) to enable membranes of the virus and host cell to fuse (Hoffmann et al., 2020; Oberfeld et al., 2020). The virus enters cells by endocytosis. The 30 kb single-stranded plus-strand RNA is released directly into the cytoplasm and hijacks the cell to translate the viral replication-transcription complex (RTC) in a double membrane vesicle. The RTC then produces RNAs that translate into protein, the ORFs coding for 16 nonstructural proteins, four main structural proteins and other special proteins (Oberfeld et al., 2020). Virions are assembled with RNA encased by nucleocapsid (N) and a “coat” consisting of membrane (M), envelope (E) and spike (S) proteins. Once released, the viral particles can infect cells in the lower airways (Type II pneumocytes) and enterocytes in the gastrointestinal tract (Bao et al., 2020; Lamers et al., 2020).

## COVID-19: Symptomatology, Clinical Disease, and Potential Therapies

Clinical symptoms of infection, although highly variable, are fever, dry and persistent cough, fatigue, anosomia/dysgeusia, loss of appetite and dyspnea but a plethora of other signs may, or may not, be present (e.g. headaches, sore throat, myalgia, rigors, intestinal discomfort/diarrhea, ocular manifestations, etc) (Guan et al., 2020; Oberfeld et al., 2020; Vetter et al., 2020). Severe symptoms leading to hospitalization that progress rapidly to hypoxia and respiratory distress requiring supplemental oxygen and ventilator support are most prevalent in the elderly with underlying comorbidities (Du et al., 2020). However, an unusual presentation in children, similar to Kawasaki Disease, termed MIS-C (multisystem inflammatory disease in children) is emerging (Viner and Whittaker, 2020). Why many viral-positive individuals are asymptomatic, or exhibit only minor cold symptoms, remains incompletely understood. Increasingly clear is the multifocal nature of COVID-19 pathogenesis with SARS-CoV-2 sometimes instigating destruction to blood vessel endothelial cells leading to coagulopathy and strokes, as well as potential kidney and neurological problems (Sardu et al., 2020). Blood sampling in moderate-severe cases may reveal lymphocytopenia, elevation of inflammatory markers like C-reactive protein (CRP) and the cytokine interleukin 6 (IL-6), along with a pro-coagulant state exhibiting elevated D-dimer (a fibrin-degradation product), indicative of an immune response out of control called a cytokine storm (Chen G. et al., 2020). There are no therapies or preventative vaccines for our immune naïve global population. Hundreds of existing drugs and a common vaccine, repurposed for COVID-19, are undergoing clinical trials. The existing vaccine being studied in the Netherlands and Australia is for tuberculosis. BCG (Bacille Calmette-Guérin) has previously shown mixed, but broadly protective, effects against a variety of respiratory infectious diseases (O'Neill and Netea, 2020). The early hype revolved around (hydroxy)chloroquine (malaria, autoimmune indications with antiviral properties) but the drug appears to offer minimal benefit for COVID-19 resolution, with the potential for harm, and some clinical trials have been shut down (Ferner and Aronson, 2020; Tang et al., 2020). Remdesivir, an antiviral drug currently under FDA Emergency Use Authorization (EUA), has shown modest benefit reducing symptoms and recovery by approximately 4 days in one study^2^ but no effect in another (Wang Y. et al., 2020). Tocilizumab, a recombinant humanized anti-human IL-6 receptor monoclonal antibody, may be effective at rendering the cytokine storm-inducing effects of IL-6 less capable of initiating damage to the airways but more studies are required^3^. Interferon (IFN)-α2b is looking promising as early results suggest it reduces viral load in the upper airways and can reduce CRP and IL-6 (Zhou Q. et al., 2020). Drug therapy aside, a major step forward in this current pandemic is the generation of a safe and effective preventative vaccine. Several articles have already appeared on this topic (Ahn et al., 2020; Amanat and Krammer, 2020; Caddy, 2020; Callaway, 2020a; Cohen, 2020; Kim et al., 2020; Lurie et al., 2020; Wang F. et al., 2020; Wu, 2020). This article deals with a late May 2020 snapshot of the global race for a SARS-CoV-2 vaccine that is taking place at breakneck speed, with a brief prelude on diagnostics and history of vaccines.

## COVID-19 Diagnostics

Knowing the genetic makeup of SARS-CoV-2 is essential for COVID-19 diagnostics. The 30 kb SARS-CoV-2 genome sequence analysis of a patient isolate from Wuhan at the epi-center in China, published in early January 2020, took place swiftly after viral isolation (Zhou P. et al., 2020). From January-May 2020, the genome has been sequenced a multitude of times in clinical isolates obtained from patients around the world (Mavian et al., 2020). The SARS-CoV-2 genome is about 80% similar to that of SARS-CoV-1 and 50% to MERS-CoV but 96% to a bat coronavirus (Andersen et al., 2020; Zhou P et al., 2020). Since deciphering the original sequence, some genetic drift is occurring in worldwide cases with mutations even arising in the Spike-encoding region, the key gateway to host entry, which could be altering virulence (Becerra-Flores and Cardozo, 2020). Besides the four structural proteins of SARS-CoV-2 mentioned above (S, E, M, N), and the critical RTC, there are several additional open-reading frames (ORFs) encoding nonstructural proteins and a viral protease within the SARS-CoV-2 genome, some with clear functions deduced from prior study with SARS-CoV-1 but others with unknown function (Ahn et al., 2020). Interestingly, ORF3b appears to suppress strongly the important Type I cytokine host immune response by blocking interferon (Konno et al., 2020).

Reverse transcriptase-polymerase chain reaction (RT-PCR) is the hallmark laboratory diagnostic technology to detect viral nucleic acid obtained from nasal swabs. Each jurisdiction around the world has designated screening assays using primer sets from either N, E, RNA-dependent RNA polymerase, ORF1a or ORF1b sequences (Ahn et al., 2020). Reliable COVID-19 diagnostic tests to detect individuals exposed to the virus is of paramount importance. Many asymptomatic infected people and those that have recovered from COVID-19 should carry immunity to further infection (Danchin and Timmis, 2020). This includes the production of neutralizing antibodies to SARS-CoV-2 (Wang C. et al., 2020). The hunt is on for rapid detection assays for those immune-privileged individuals. Our colleagues have carried out detailed analyses of over 300 viral antigen/serological antibody tests seeking approval (or already with EUA) for this important area (Ghaffari et al., 2020).

## Generating the “Ideal” Vaccine for Protection Against COVID-19

### Timelines for Vaccine Development

**Figure 2:**
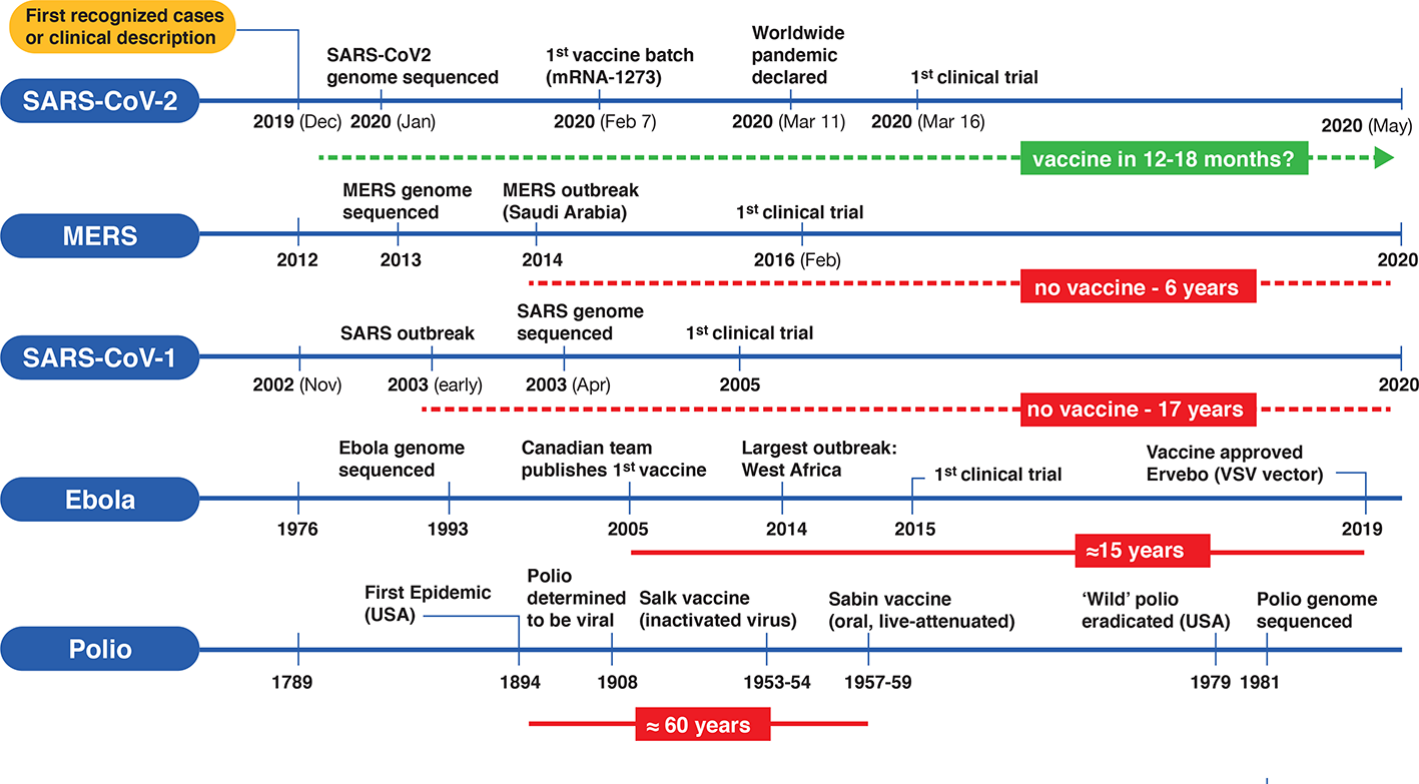
Timelines for the development of various vaccines for polio, Ebola virus, and three betacoronaviruses (SARS-CoV-1, MERS-CoV and SARS-CoV-2). On the left side of each timeline is a reference point for the first clinical case description and/or first recognized cases) for each type of viral infection. Significant events are depicted along the timeline (not according to scale). Solid horizontal red bars indicate the approximate period from first outbreak/vaccine to clinically approved use. Dotted red lines indicate no availability of vaccines since first outbreak and the green dotted line represents a rapid emergency use authorization timeline for a putative SARS-CoV-2 vaccine.

Vaccines are one of the monumental achievements in human medical intervention to mitigate the dispersion and impact of infectious disease. Polio was a serious crippling disorder for decades until the development of two separate vaccine candidates by Jonas Salk and Albert Sabin (Melnick, 1996). From the time of the first polio outbreak in the United States to testing and development was almost 60 years^4^ (**Figure 2**). More recently, a vaccine for the lethal infectious hemorrhagic fever elicited by Ebola disease took on a substantially different timeline with development by a Canadian team (Jones et al., 2005) preceding the worst outbreak in Western Africa in 2014, with the disease first described in isolated cases in 1976. Taking into account the prolonged development and testing phases it still took at least 15 years (**Figure 2**) for authorized clinical use of an Ebola vaccine^5^. Looking at the other coronavirus disease-causing outbreaks (SARS and MERS) that led to regional epidemics in various countries, the impetus was strong initially to develop vaccines but fizzled out with their spontaneous resolution. Although vaccine candidates were brought forward for both SARS-CoV-1 and MERS, and projects continue, there are still no approved vaccines for either infectious agent 17 and 6 years, respectively (de Wit et al., 2016; Song et al., 2019), since the original outbreaks (**Figure 2**). In view of this, the bar is set high for developing an effective vaccine targeting COVID-19. Will SARS-CoV-2 go the same direction as the other coronaviruses, or will it become a seasonal outbreak like influenza and persist as a moderately benign to severe nuisance-causing infectious disorder that lasts for years?

### Current Context for COVID-19 Vaccine Development

Given the worldwide magnitude of the COVID-19 pandemic and comparisons to the great Spanish flu of 1918, the race for a vaccine candidate has taken on unprecedented urgency and commitment across the globe. Established organizations are maintaining databases of vaccines under development including the World Health Organization^6^ (WHO), the Coalition for Epidemic Preparedness Innovations^7^ (CEPI), the Milken Institute^8^ (a nonprofit think-tank out of California), and Biocentury Inc^9^ (a partner to the biopharmaceutical industry). Unparalleled data sharing and collaborative team efforts are breaking down barriers in an attempt to reduce the time from the usual 10^+^ years for an approved vaccine down to 12–18 months. CEPI, the Biomedical Advanced Research and Development Authority^10^ (BARDA), GAVI, The Vaccine Alliance^11^ (formerly Global Alliance for Vaccines and Immunization), various governments and other sources are either pouring money into the efforts to fund projects or providing logistical support with additional initiatives. One such initiative is the strategic alliance ACTIV^12^ (Accelerating COVID-19 Therapeutic Interventions and Vaccines), a public-private initiative bringing together more than a dozen leading biopharmaceutical companies, the CDC (Centers for Disease Control and Prevention), FDA (U.S. Food and Drug Administration), and EMA (European Medicines Agency) to develop an international strategy for a coordinated research response to the COVID-19 pandemic. Another massive undertaking to expedite the process is Operation Warp Speed^13^, which includes scientists, pharmaceutical companies and US federal officials and is being compared to the Manhattan Project^14^

**Figure 3:**
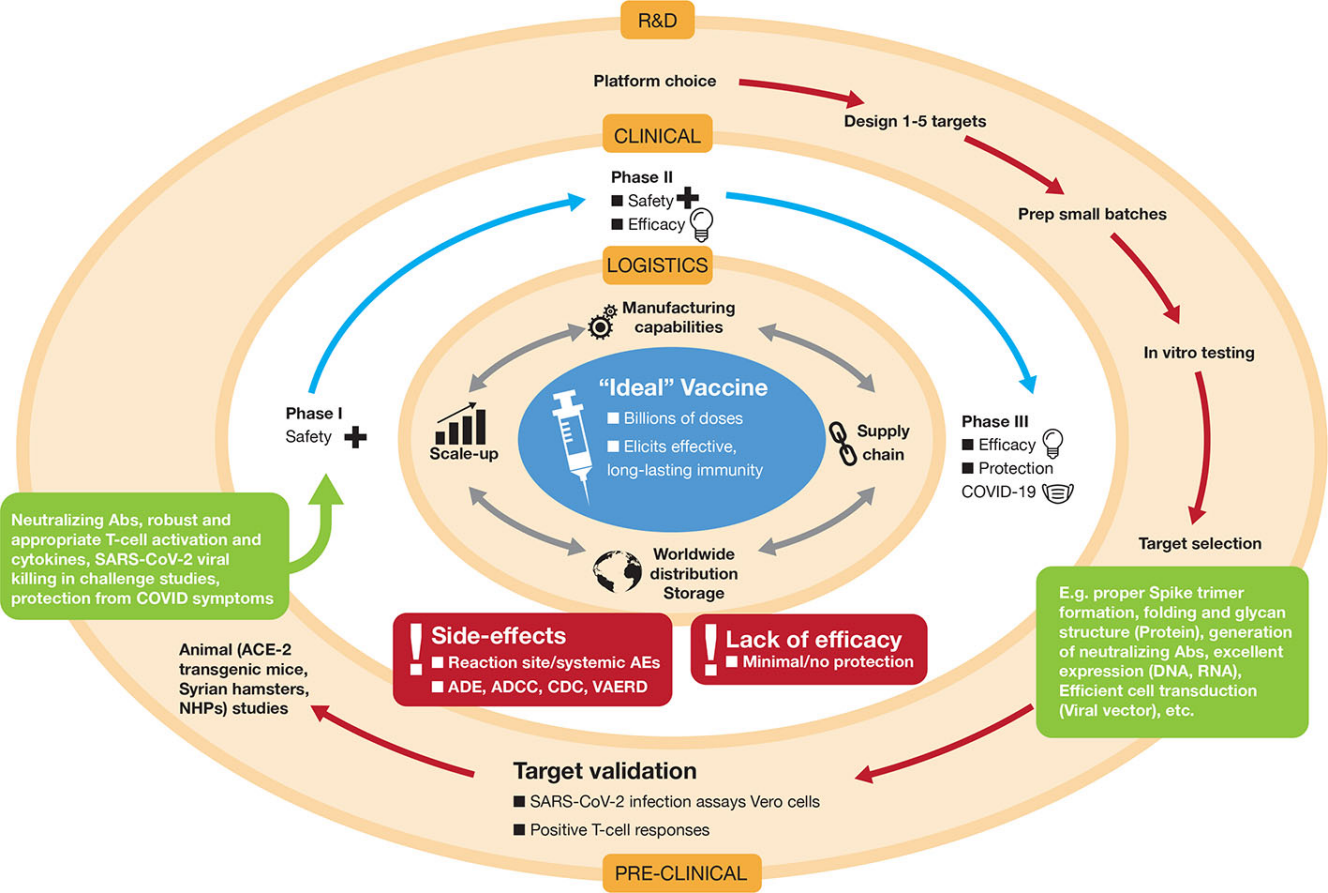
Development of the “ideal” vaccine for COVID-19 depicted by concentric circles converging to the target. Normally, development occurs in three discrete phases: Research and Development (R&D) involving platform selection, designing targets (e.g. whether that might be selection of an RNA sequence and decisions on nucleoside substitutions, lipid nanoparticle (LNP) formulation, etc, or decisions on how to create a live-attenuated viral preparation) and preclinical testing *in vitro* in cell culture and *in vivo* in animals. For SARS-CoV-2 R&D, some of the animal models used are transgenic mice that overexpress the Spike-binding protein ACE-2, Syrian hamsters, ferrets, and non-human primates (NHPs). If encouraging results are apparent in the preclinical phase (indicated by various parameters in green boxes), the candidate vaccine is taken to the second phase, which consists of testing in human volunteers in three stages of clinical trials (Phase I, Phase II, and Phase III). These may be concatenated to expedite approval (e.g. Phase II/III). Due to the pandemic nature of COVID-19, both these outer concentric phases are being pursued simultaneously under expedited approvals with potential for emergency use authorizations. If, and only when, vaccine safety and efficacy is achieved in human volunteers, the logistical operations become the major hurdles to ensure worldwide distribution in a coordinated and inter-connected manner (manufacturing, supply chain distribution, storage, etc.). Vaccine candidates that do not achieve satisfactory results in clinical trials, due to various factors shown in the red boxes, will be dropped from further development. ADE, antibody-dependent enhancement; ADCC, antibody-dependent cellular cytotoxicity; CDC, complement-dependent cellular cytotoxicity; VAERD, vaccine-associated enhanced respiratory disease; AE, adverse event.

What does the “ideal” vaccine look like for COVID-19 prevention? In clinical terms, three main factors are essential: (i) a robust immune response generating long-lasting *neutralizing antibodies to SARS-CoV-2* antigens (e.g. S and/or N) is imperative (**Figures 1** and **3**). When individuals are infected with foreign antigens on viruses, they evoke both innate and adaptive immune reactions with a coordinated antigen-presenting cell (APC) attack on the virus and T-helper cell activation that leads to B-lymphocytes producing antibodies. In this context, ideally the antibodies will directly interfere with SARS-CoV-2's knack for latching onto epithelial cells and Type II pneumocytes *via* ACE2 (**Figure 1**) in order to neutralize the virus. This would curtail successfully the virus from infecting the host and be protective if an infection resulted. However, there remains critical shortfalls in the current knowledge of what comprises a protective immune response against COVID-19 and how long it lasts [as of May 2020]. (ii) The ideal SARS-CoV-2 vaccine will also induce *potent T-lymphocyte immunity*. Ideally, this would be a well-coordinated, orchestrated T-cell response that includes T-helper and cytotoxic T-lymphocyte subsets that recognize SARS-CoV-2 infected cells in the body and annihilate them to block viral replication, along with acquisition of memory T-cells to prevent reinfection months to years later. (iii) The candidate vaccine should *limit any serious adverse events (SAEs)* at the injection site or systemically, for example, fever in infants and young children. In the case of respiratory disorders caused by infectious agents, it is essential that vaccine-associated enhanced respiratory disease (VAERD), antibody-dependent enhancement (ADE), antibody-dependent cellular cytotoxicity (ADCC), complement-dependent cytotoxicity, including cytokine storm-inducing effects are completely avoided (Graham, 2020; Hotez et al., 2020; Vardhana and Wolchok, 2020). Some of these clinical sequelae have been associated with past vaccines in development (**Figure 3**), with a prominent example that for respiratory syncytial virus (RSV) during the late 1960s (Graham, 2020). Since the virus apparently activates complement deposition in small blood vessels of some infected individuals (Magro et al., 2020), blocking adverse host responses is essential. Complement, inflammation, and coagulation systems are intertwined (Oikonomopoulou et al., 2012) and hyperactive in COVID-19 positive cases and the vaccine should not provoke these host response systems.

In logistical terms, there are a few essential parameters for the ideal candidate vaccine for COVID-19: (i) the vaccine should be *easy to administer and preferably in a single dose* at the lowest possible amount. An oral or intranasal vaccine would be ideal. (ii) The vaccine should be *facile to produce and scale-up*. Manufacturing millions to billions of doses required to immunize the human population must be feasible and rapid. (iii) *Long-term storage* of the vaccine at room temperature should be a sought after goal to facilitate transport and stockpiling in underdeveloped nations with inadequate supply chains and cold chain capacities (**Figure 3**). The ideal vaccine targeting COVID-19 would fit into a proposed target product profile as described in **Table 1**.

**Table 1:** Target product profile of “ideal” SARS-Cov-2 vaccine targeting COVID-19.

Indication and Clinical Use
Prophylactic vaccination against SARS-CoV-2 infection of susceptible health-care workers (HCW), children, adolescents and adults.
**Use-case scenarios**
For use in (current) pandemic response and subsequent wave outbreaks. For use in routine immunization, supplementary immunization and stockpiling of vaccine against COVID-19.
**Formulation**
Contains SARS-CoV-2 vaccine in sterile liquid (or other format) for direct oral use or in lyophilized format with simple reconstitution at point of use not requiring end-to-end cold chain.
**Presentation**
Single-use or multidose vial liquid or lyophilized, stored at room temperature.
**Stability**
Thermostable, not light sensitive. The stability of vaccine potency is durable >2 yrs at room temperature.
**Dose regimen and amount**
Single dose 0.5 ml or less, if injectable. A second boosting dose 4-8 weeks later, if necessary.
**Route of administration**
Oral (preferred). Subcutaneous, intradermal, or intramuscular (acceptable).
**Target population**
All individuals aged 9 months and above.
**Target countries**
All countries around the world.
**Safety**
The safety profile should be based on at least two data sets with preferably at least 10,000 subjects over all age groups.
**Immunogenicity**
Phase III clinical studies should include an immunogenicity arm in which serum and blood samples are collected and tested for antibody and cell-mediated immunity. If possible, a minimum serological correlate of protection should be determined based on the short-term and long-term efficacy.
**Interactions**
The SARS-CoV-2 vaccine should not be administered with any other vaccine or it can be administered concomitantly with influenza vaccine at separate sites.
**Pregnancy**
There is limited information on administration of SARS-CoV-2 vaccine to pregnant or lactating women.
**User training requirements**
Minimal training administered by HCW or trained layperson.
**Cost/immunized individual**
COGS should be reasonable to governments of both high- and low-income nations.
**Disposal**
Safe disposal of vials/sharps in biohazard waste at point of use.

### The Traditional Process Towards Vaccine Development

The normal strides taken to achieve successful vaccine development are similar to those for any drug. The process should be very stringent seeing that it will culminate in administration of the vaccine candidate to billions of humans. The typical paradigm is depicted in **Figure 3** and is explained in the legend.

### The Platforms Currently Exploited for SARS-CoV-2 Vaccine Development

**Figure 4:**
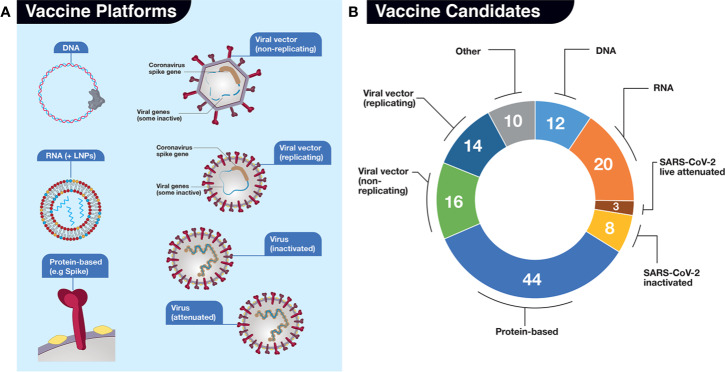
Vaccine candidates in development for SARS-CoV-2. **(A)** For our analyses, we have divided potential vaccines into 7 (seven) main platforms (DNA, RNA, Protein-based, Viral vector-based (non-replicating), Viral vector-based (replicating), Virus (inactivated), and Virus (live, attenuated). An additional “Other” category for those platforms where there is uncertainty from described sources is also included but not shown. The platforms are represented by various simple drawings. LNPs, lipid nanoparticles. **(B)** Numbers of vaccine candidates in development late May 2020, depicted in pie chart format, in each platform. Sources include: The Milken Institute, WHO, BioCentury, and our own investigations. Categorization of platforms differs slightly in each source.

The scientific publications to date (Ahn et al., 2020; Amanat and Krammer, 2020; Caddy, 2020; Callaway, 2020a; Callaway, 2020b; Cohen, 2020; Chen WH et al., 2020; Kim et al., 2020; Lurie et al., 2020; Thanh Le et al., 2020; Wang F et al., 2020; Wu, 2020), as well as databases mentioned above, have various formats for classifying vaccine platforms. In our analysis, we consider seven main platforms of vaccine development, along with an eighth catch-all “Other” category (**Figure 4**). There are two nucleic acid platforms: *DNA* (12 candidates) and *RNA* (20^+^ candidates), which could be sub-divided further according to particular traits related to delivery and carriers (e.g. electroporation with special devices intradermally vs oral formulation or LNPs vs exosomes) but for simplicity are considered only as two categories. These nucleic acid platforms belong to the new generation of vaccines. Neither has reached licensing for human use but a number are being tested in humans (Cohen, 2020). A third category termed “*Protein-based*” (also referred to as “subunit” vaccine) includes a broad range of technologies to prepare immune-stimulating viral protein antigens and represents the largest category of all current COVID-19 vaccine candidates (currently 44^+^) in development. Like nucleic acids, protein-based vaccines represent a newer technology but some are already in use in the clinical realm (e.g. Gardasil for human papillomavirus). The fourth and fifth categories are *viral-based vectors*, similar to those used in gene therapy and include *nonreplicating* (16^+^ candidates) and *replicating* (14^+^ candidates) vectors. The next two are the *SARS-CoV-2 viruses* themselves, either *inactivated* (usually with a chemical such as β-propriolactone, which chemically inactivates enveloped viruses and can inhibit viral membrane fusion in a dose-dependent manner)(Gao et al., 2020) or in a *live-attenuated* version, generated by techniques such as codon deoptimization or serial passaging in cell culture (Mueller et al., 2020). The “*Other*” category includes virus-like particles (VLPs), the use of non–SARS-CoV-2 virus carriers such as killed rabies (CORAVAX) and live modified horsepox (TX-1800). There are 10^+^ in this category. Repurposed existing vaccines for polio or tuberculosis to evoke general immunity, various cellular immunotherapies to stimulate the host immune system, encapsulated convalescent serum, and “unknown” platform designation (listed in The Milken Institute database) are excluded from our analyses (**Figure 4**). According to these criteria, there are over 125 SARS-CoV-2 vaccines in development, which is astounding, from all across the globe in such a short time-frame.

### Advantages and Disadvantages of Vaccine Development Platforms

For the ideal COVID-19 vaccine to reach routine use in humans, it is imperative that the vaccine protects against both clinical disease and viral transmission, in order to break the chain of person-to-person pandemic spread. Some of the advantages/disadvantages of each platform are considered in **Table 2**.

**Table 2:** Advantages/disadvantages of each vaccine platform.

	Advantages	Disadvantages
**DNA**	No handling of infectious material Can be used in immunocompromised subjects Reasonable and enduring immune response, both humoral and cell-mediated No anti-plasmid immune responses allowing for effective boosting (reported by Inovio) Rapid and scalable manufacturing Long-term stability Options for multivalent formulation Oral formulation possible (Symvivo)	No approved DNA vaccines, along with real-world experience in the global population Variable mucosal immunity and other immune responses Some require special tools for delivery to the sub-dermal layer (Inovio) Potential risk for host cell genomic integration (though likely small for plasmids)
**RNA**	Rapid design and production No handling of infectious material No potential for insertional mutagenesis Strong early antiviral responses, both humoral and cell-mediated Options for multivalent formulation Scaling up to global-wide production appears feasible but not yet tested	No approved RNA vaccines, but some clinical testing for other viruses (rabies, influenza) Inflammatory reactions possible Most formulations require a cold chain for longevity and stability Ubiquitous ribonucleases require careful design with substituted nucleosides and skilled formulation of lipid nanoparticle carriers for effective delivery Boosting likely necessary to achieve robust and long-lasting immunity
**Protein**	No handling of infectious material Strong antibody responses Precedent for successful vaccines of this platform Viral protein complexes can be formulated to simulate virus patterning (VLPs)	Need for adjuvants Scale up of manufacturing can be challenging Potentially lacking correct glycan shield of native virus
**Viral Vector (both replicating/non-replicating)**	Years of experience in the gene therapy field studying safety, immune responses Strong antibody and cellular responses	Risk for chromosomal integration and oncogenesis Cannot be used in immunocompromised subjects Preexisting antibodies to some vectors possible Potential for inflammatory AEs Variable immunogenicity Significant manufacturing hurdles at scale
**Live Virus (attenuated)**	Proven technology Strong immune response Multivalent Simple formulation, not requiring adjuvants Proven track record for economical large-scale manufacturing	Requires dedicated biosafety level facilities Risk for attenuated virus to regain virulence Can be complicated to scale up manufacturing
**Virus (inactivated)**	Proven technology Strong immune response Multivalent Simple formulation, not requiring adjuvants	Requires dedicated biosafety level facilities Complicated to scale up manufacturing

This is by no means an exhaustive list of pros/cons. There exists an enormous challenge for bringing forward an ideal vaccine to treat SARS-CoV-2 infection.

### Representative COVID-19 Vaccines

**Figure 5:**
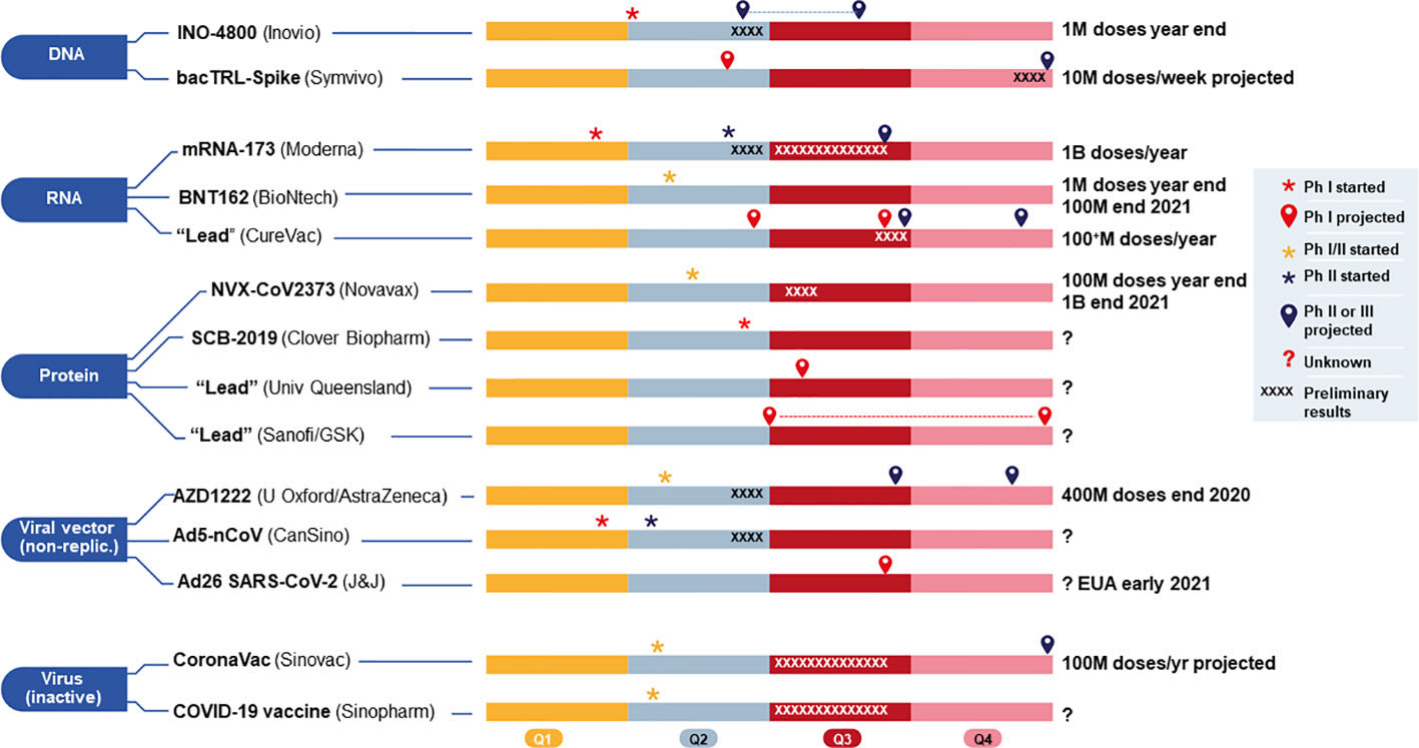
Current 2020 and projected timelines (shown in four quarters, Q1–Q4) for 14 vaccine candidates, grouped by platform, either funded by (CEPI/BARDA, and currently in (or approaching) clinical development for COVID-19. The two candidates using viral vector (replicating) from Pasteur/Themis (Merck) and live attenuated virus (Univ Hong Kong) funded by CEPI, covered in the main text body, are not included here because they are still too early in development and without sufficient information. M, million; B, billion; Ph, Phase. Sources include publicly available company websites.

In this section, we provide some specific details on 16 (of >125) vaccines in the pipeline that are registered for clinical trials or already being tested (as of May 25, 2020), in addition to those that are currently CEPI- and/or BARDA-sponsored from each platform that are, or not yet, in clinical trials. This is to provide a snapshot of the field in early endeavors to procure a functional SARS-CoV-2 vaccine in man and their proposed developmental pipeline (**Figure 5**). One can also refer to **Table 3** for an abbreviated account of the 16 candidates. Where the vaccine candidate's name has not been specified, we refer to it as “Lead.” We do not endorse any particular candidate. CEPI/WHO/BARDA/GAVI and governments are taking the pan-platform approach, recognizing that this is the best path forward.

**Table 3:** Some SARS-CoV-2 vaccine candidates in the pipeline in (or nearing) clinical trials.

Platform	Vaccine Name	Company/Research Group/Partners	Target	Stage
DNA	INO-4800	**Inovio** Pharmaceuticals; CEPI; Beijing Advaccine Biotechnology (China); International Vaccine Institute (Korea); VGXI Inc.; Ology Bioservices; Richter-Helm BioLogics	Spike	Phase I
DNA	bacTRL-Spike	**Symvivo Corporation**	Spike	Phase I (not started yet)
RNA	mRNA-1273	**Moderna**/NIH-NIAID; CEPI; BARDA; Lonza; Operation Warp Speed	Spike	Phase I/II
RNA	BNT162 (a1, b1, b2, c2)	**BioNTech SE/Pfizer;** Fosun Pharma (China); Operation Warp Speed	3CLpro, NSP5, Mpro, other	Phase I/II
RNA	“Lead”	**CureVac** AG/Paul Ehrlich Institute; CEPI	Undisclosed	Phase I/IIa (summer 2020)
Protein-based	NVX-CoV2373	**Novavax**; CEPI; Emergent BioSolutions; Praha Vaccines; Serum Institute of India	Spike (prefusion)	Phase I/II
Protein-based	SCB-2019	**Clover Biopharmaceuticals**; Clover Australia; CEPI; GSK; Dynavax; Chengdu Hi-Tech Park; Chengdu Clinical Center for Public Health	Spike trimer	Phase I (June 2020)
Protein-based	“Lead”	**University of Queensland**; CEPI; GSK; Dynavax; Cytiva (previously GE Healthcare Life Sciences); Lonza;, Thermo Fisher Scientific; CSL/Seqirus	Spike trimer	Preclinical; Phase I not started
Protein-based	“Lead”	**Sanofi and** GSK; BARDA	Spike	Preclinical; Phase I projected last half 2020
Viral vector (non-replicating)	AZD1222 (ChAdOx1 nCoV-19)	**University of Oxford (Jenner Institute)/Astra Zeneca** CEPI; Vaccitech Ltd; Serum Institute of India; WuXi Biologics Inc.; Vaccines Manufacturing and Innovation Centre (Pall Life Sciences, a unit of Danaher Corp.); Cobra Biologics AB; Dutch CDMO Halix B.V.; Merck; Oxford Biomedica plc; Advent Srl; Operation Warp Speed	Spike	Phase I/II, Phase II/III starting soon
Viral vector (non-replicating)	Ad5-nCoV	**CanSino Biologics**; Beijing Institute of Biotechnology, Academy of Military Medical Sciences; PLA of China Jiangsu Province Centers for Disease Control and Prevention; Hubei Provincial Center for Disease Control and Prevention, Tongji Hospital; Jiangsu Provincial Center for Disease Control and Prevention; Canadian Immunization Research Network at the Canadian Center for Vaccinology	Spike	Phase II
Viral vector (non-replicating)	Ad26 SARS-CoV-2	**Johnson & Johnson – Janssen**; BARDA; Emergent BioSolutions Inc.; Beth Israel Deaconess Medical Center; Catalent; Operation Warp Speed	Undisclosed	Preclinical; Phase I projected Sept 2020
Viral vector (replicating)	“Lead”	**Institut Pasteur – Themis/Merck**; CEPI, University of Pittsburgh; ABL Europe	“selected, incorporated protein antigens from SARS-CoV-2”	Preclinical
Virus (inactivated)	CoronaVac (PiCoVacc)	**Sinovac Biotech**/Sinovac R&D Co; Dynavax	Whole virus (Spike RBD main immunogen)	Phase I/II
Virus (inactivated)	COVID-19 vaccine	China National Pharmaceutical Group, **Sinopharm**; Wuhan Institute of Virology (WIV)	Whole virus	Phase I/II
Virus/Other (attenuated)	“Lead”	**University of Hong Kong**; CEPI	Undisclosed SARS-CoV-2 proteins	Preclinical

Sources include: WHO, CEPI, The Milken Institute, the COVID-19 Resource Center of Biocentury^6-9^

DNA Vaccines

**(i) Name:** INO-4800 (Smith et al., 2020) **Company (Country):** Inovio (USA)

**Antigen**: SARS-CoV-2 Spike glycoprotein (S)

**Details of platform**: DNA plasmid encoding S delivered by CELLECTRA^®^ 2000 electroporator

**Route/mode of administration**: intradermal injection followed by electroporation

**Safety of Platform**: Inovio's method has been tested in >2,000 people with >6,000 administrations with a favorable safety and tolerability profile. However, DNA vaccines are not currently in use in humans

**Advantages**: fast design/manufacturing; no cold chain for storage/distribution; robust immune response

**Clinical Trial Registration**: NCT04336410 **Start date**: April 6, 2020

**Official Name of Trial**: Safety, Tolerability and Immunogenicity of INO-4800 for COVID-19 in Healthy Volunteers

**Study design**: Phase I, non-randomized open label with Groups 1 and 2 for 18-50 y.o, n=40 at two clinics (in US); **Dosing Regimen**: Single 1 mg dose of INO-4800 followed by electroporation using the CELLECTRA^®^ 2000 device (Group 1) and dual injections of 1 mg (total 2 mg) of INO-4800 followed by EP using the CELLECTRA^®^ 2000 device per dosing visit at Days 0 and 28; **Endpoints**: Primary – General safety including percentage with adverse events (AEs) and local injection site reactions up to 52 weeks; Ag-specific binding titer and IFN-γ increase from baseline

**Partners**: Beijing Advaccine Biotechnology (China) and International Vaccine Institute (Korea)

**Funding Partners:** Coalition for Epidemic Preparedness Innovations (CEPI), the Bill & Melinda Gates Foundation, and the US Department of Defense

**Manufacturing partners:** VGXI, Inc., Richter-Helm, and Ology Bioservices

**Next stage**: Preliminary data end June 2020; starting Phase II/III late summer/Fall 2020

**Timeline**: 1 million doses of INO-4800 by the end of 2020 (pending regulatory approval)

**(ii) Name:** BacTRL-Spike (a trivalent version is in development) **Company (Country):** Symvivo (Canada)

**Antigen**: SARS-CoV-2 Spike glycoprotein (S)

**Details of platform**: DNA plasmid expressing trimeric S and a hybrid transporter protein within *Bifidobacterium longum* delivered to colonic epithelial cells to prime an immune response *via* colonic lymphoid tissues

**Route/mode of administration**: oral ingestion of probiotic capsule

**Safety of Platform**: Extensive worldwide use of probiotics. However, DNA vaccines are not currently on the market for use in humans and this particular strategy is untested.

**Advantages**: fast design and manufacturing; no cold chain for storage and distribution; robust immune response and mucosal immunity predicted

**Clinical Trial Registration**: NCT04334980 **Start date**: June 2020 (projected)

**Official Name of Trial**: A Phase 1, Randomized, Observer-Blind, Placebo-Controlled Trial to Evaluate the Safety, Tolerability and Immunogenicity of the bacTRL-Spike Oral Candidate Vaccine for the Prevention of COVID-19 in Healthy Adults

**Study design**: Phase I, dose-escalating with three cohorts, n=84; **Dosing Regimen**: Single oral dose of BacTRL-Spike at three doses (1, 3, or 10 million bacteria) or placebo in each cohort (with n=63 for vaccine and n=21 for placebo); **Endpoints**: Primary – Incidence and severity of adverse events (AEs) Secondary: Seroconversion, virus stool shedding, protection from COVID-19

**Partners**: none announced

**Funding Partners:** none announced

**Manufacturing partners:** none announced

**Next stage**: Obtain results of Phase I study by end of the year and get 10,000^+^ enrolled in further Phase II/III studies

**Timeline**: 5000 doses by July 2020 and goal of 10 million doses/week through manufacturing scale up

RNA Vaccines

**(i) Name:** mRNA-1273 (13 candidates in development) **Company (Country):** Moderna (USA)

**Antigen**: Full-length prefusion stabilized SARS-CoV-2 Spike glycoprotein (S)

**Details of platform**: Non-replicating RNA genetic material mixed with LNP formulation (proprietary ionizable lipid, SM-102, and three commercially available lipids, cholesterol, DSPC, and PEG2000 DMG)

**Route/mode of administration**: i.m. deltoid

**Safety of Platform**: >1500 injections with Moderna's RNA formulations for Zika, RSV, CMV, Chikungunya viruses have taken place in other Phase I and II clinical trials and are generally well-tolerated. No RNA vaccines are currently licensed and approved for use in humans

**Advantages**: fast design and manufacturing; robust immune response

**Clinical Trial Registration**: NCT04283461 (NCT04405076) **Start date**: March 16, 2020 (IND for Phase II approved May 7 and started early June 2020)

**Official Name of Trial**: Safety and Immunogenicity Study of 2019-nCoV Vaccine (mRNA-1273) for Prophylaxis of SARS-CoV-2 Infection (COVID-19)

**Study design**: Phase I, non-randomized open label with Arms 1–3 for 18–54 y.o., n=15/arm, Arms 4–6 for 55–70 y.o., n=10/arm and Arms 7–9 for 71^+^ y.o., n=10/arm; **Dosing Regimen**: Dual 25, 100, or 250 μg in 0.5 ml on Days 1 and 29 followed up to 12 months after 2^nd^ injection; **Endpoints**: Primary – General safety including local reactogenicity and serious adverse events (SAEs) throughout the study duration; Secondary - mean increase general Ab and IgG titers from baseline, percentage subjects with seroconversion at Day 57

**Partners**: NIAID/NIH; Operation Warp Speed

**Funding Partners:** CEPI, BARDA

**Manufacturing partners:** Lonza

**Next stage**: Obtain complete results of Phase I (see interim results below) and get 10,000^+^ enrolled in Phase II/III studies

**Timeline**: scaling up manufacturing capacity toward the production of millions of doses per month, in the potential form of individual or multidose vials, with goal of 1 billion doses/year

**(ii) Name:** BNT162 **Company (Country):** BioNtech SE (Germany)/Pfizer (USA)

**Antigens**: SARS-CoV-2 3C-like protease (3CLpro), NSP5, SARS-CoV-2 main protease (Mpro)

**Details of platform**: Uridine containing mRNA (uRNA) or nucleoside modified mRNA (modRNA) mixed with proprietary lipoplex formulation for the three antigens, as well as a fourth vaccine candidate self-amplifying mRNA (saRNA)

**Route/mode of administration**: i.m. injection (0.5 ml) prime and boost

**Safety of Platform**: No RNA vaccines are currently licensed and approved for use in humans

**Advantages**: fast design and manufacturing; robust immune response

**Clinical Trial Registration**: NCT04368728 (also NCT04380701; 2020-001038-36) **Start date**: April 29, 2020

**Official Name of Trial**: Study to Describe the Safety, Tolerability, Immunogenicity, and Potential Efficacy of RNA Vaccine Candidates against COVID-19 in Healthy Adults

**Study design**: Phase I/II, randomized, placebo-controlled, observer-blind, dose-finding, and vaccine candidate-selection study in healthy adults. 18 experimental arms each with four vaccine targets (BNT162a1, BNT162b1, BNT162b2, BNT162c2) x 3 age groups (18–55 y.o., 65–85 y.o. and 18–85 y.o.) × 2 dosing time points (timing not specified) × 3 doses (low-, mid-, high-; amounts not specified) + 3 placebo comparator arms carried out in three stages; Stage 1: to identify preferred vaccine candidate(s), dose level(s), number of doses, and schedule of administration (with the first 15 participants at each dose level of each vaccine candidate comprising a sentinel cohort); Stage 2: an expanded-cohort stage; and Stage 3; a final candidate/dose large-scale stage; n=7400; **Dosing Regimen**: Single and dual doses to be tested; **Endpoints**: Primary – General safety, incidence and severity of adverse events (AEs) up to a week after each dose and blood chemistry in sentinel cohort; Secondary: Various immune parameters and incidence of COVID-19

**Partners**: Fosun Pharma (clinical trials in China); Operation Warp Speed

**Funding Partners:** Pfizer

**Manufacturing partners:** Pfizer (3 sites in USA and 1 in Belgium)

**Next stage**: Move quickly through Phase I and recruit up to 8000 volunteers for Phase II

**Timeline**: Millions of doses by year end (for clinical trials in China) and hundreds of millions by end of 2021

**(iii) Name:** “Lead candidate” **Company (Country):** CureVac AG (Germany)

**Antigen**: undisclosed

**Details of platform**: Engineered RNA genetic material mixed with LNPs (from either Acuitas or Arcturus) or proprietary CureVac Carrier Molecule

**Route/mode of administration**: presumably i.m. injection

**Safety of Platform**: No RNA vaccines are currently licensed and approved for use in humans but their platform has been tested for vaccine development directed toward other viruses

**Advantages**: fast design and manufacturing; robust immune response

**Clinical Trial Registration**: not yet registered **Start date**: June 2020 (projected)

**Study design**: Phase I in two European sites; **Dosing Regimen**: Single and dual doses to be tested; **Endpoints**: Initial immunogenicity and safety results

**Partners**: Paul Ehrlich Institute

**Funding Partners:** CEPI $8.3M and the EU has promised 80M Euro for scale-up and production; Bill and Melinda Gates Foundation; Defense Advanced Research Projects Agency

**Manufacturing partners:** not announced

**Next stage**: Start Phase I studies in summer 2020 and Phase II projected for Fall 2020

**Timeline**: up to several hundred million doses of bulk RNA/year with their current GMP III facility; a new GMP IV suite will be put into operation within two years where capacity for production of one billion or more doses per year is possible

Recombinant Protein-Based Vaccines

**(i) Name:** NVX-CoV2373 **Company (Country)**: Novavax (USA)

**Antigen**: Recombinant SARS-CoV-2 Spike protein (S) in prefusion state

**Details of platform**: Recombinant protein is expressed in genetically engineered Sf9 insect cells and the properly folded and post-translationally modified protein is incorporated into a nanoparticle formulation along with Novavax's saponin-based Matrix-M adjuvant

**Route/mode of administration**: i.m. injection to deltoid muscle

**Safety of Platform**: Novavax's platform has been tested in several Phase I, II, III trials for seasonal influenza, Ebola and RSV and appears to be safe

**Advantages**: fast design and relatively rapid production possible

**Clinical Trial Registration**: NCT04368988 Start date: May 15, 2020

**Official Name of Trial**: A 2-Part, Phase I/II, Randomized, Observer-Blinded Study to Evaluate the Safety and Immunogenicity of a SARS-CoV-2 Recombinant Spike Protein Nanoparticle Vaccine (SARS-CoV-2 rS) with or without MATRIX-M™ Adjuvant in Healthy Subjects

**Study design**: Phase I clinical trial (starting in Australia) is a placebo-controlled observer blinded study of about 130 healthy adults followed by Phase II in multiple countries; **Dosing Regimen**: Single and dual doses to be tested at Day 0 and 21, either 5 or 25 μg with 50 μg Matrix M; **Endpoints**: include assessment of dosage amount and number of vaccinations, along with preliminary immunogenicity and safety results

**Partners**: Emergent BioSolutions

**Funding Partners**: CEPI $388 million

**Manufacturing partner**: Emergent BioSolutions; Praha Vaccines and Serum Institute of India

**Next stage**: Preliminary data from Phase I in July, then proceeding to Phase II (2200 volunteers in multiple countries) later in summer with results expected by the end of 2020

**Timeline**: 100 million doses by year-end and a billion by end of 2021

**(ii) Name:** SCB-2019 **Company (Country):** Clover Biopharmaceuticals Inc. (China)

**Antigen**: Recombinant trimeric SARS-CoV-2 Spike protein (S)

**Details of platform**: patented Trimer-Tag^®^ technology to produce a S-Trimer protein subunit vaccine that resembles the native trimeric viral spike *via* a rapid mammalian cell-culture based expression system

**Route/mode of administration**: i.m. injection to deltoid muscle

**Safety of Platform**: Clover has previously developed recombinant subunit-Trimer vaccines for RSV and Influenza viruses utilizing its Trimer-Tag^®^ technology and has demonstrated that they are able to evoke protective neutralizing antibody responses in multiple animal models and appears to be safe.

**Advantages**: fast design and relatively rapid production possible

**Clinical Trial Registration**: NCT04405908 **Start date**: June 20, 2020 (projected)

**Official Name of Trial**: SCB-2019 as COVID-19 vaccine

**Study design**: Phase I, Randomized, double blind, placebo controlled, n=150, SCB-2019 alone or with two different adjuvants; **Dosing Regimen**: twice (Day 1 and Day 22) at three doses (3, 9, or 30 μg); **Endpoints**: includes assessment of dosage amount, number of vaccinations, along with preliminary immunogenicity and safety results

**Partners**: Chengdu Hi-Tech Park (R&D) and Chengdu Clinical Center for Public Health/Clover Australia (Clinical trials); both GSK and Dynavax (Adjuvants)

**Funding Partners:** CEPI ($3.8 M)

**Manufacturing partner:** Clover has one of the largest in-house, commercial-scale cGMP biomanufacturing capabilities in China and is partnering with GSK to use their Pandemic adjuvant System and Dynavax for their (TLR9 agonist) CpG 2018 adjuvant (the adjuvant used in HEPLISAV-B^®^ [Hepatitis B Vaccine (Recombinant), Adjuvanted], an adult hepatitis B vaccine approved by FDA)

**Next stage**: Get clinical trials underway and evaluate data

**Timeline**: not disclosed yet

**(iii) Name**: “Lead candidate” **Organization (Country):** University of Queensland (Australia)

**Antigen**: Recombinant trimeric SARS-CoV-2 Spike protein (S)

**Details of platform**: molecular clamp platform by synthesizing viral surface proteins, which attach to host cells during infection, and “clamping” them into shape, making it easier for the immune system to recognize them as the correct antigen

**Route/mode of administration**: presumably i.m. injection

**Safety of Platform**: unknown details for this specific platform

**Advantages**: fast design and relatively rapid production possible

**Clinical Trial Registration**: not yet registered **Start date**: July 2020 projected

**Official Name of Trial**: not yet started but will take place in Australia

**Study design**: Phase I not started yet; **Dosing Regimen**: not known; **Endpoints**: not known

**Partners**: Peter Doherty Institute for Infection and Immunity, Melbourne (Vaccine development), Viroclinics Xplore (Preclinical animal studies), GSK (Adjuvant)

**Funding Partners:** CEPI $10.6 M

**Manufacturing partner:** Cytiva (previously GE Healthcare Life Sciences), as well as Lonza and Thermo Fisher Scientific; CSL/Seqirus, Dynavax and GSK will supply adjuvants

**Next stage**: Get clinical trials underway

**Timeline**: not disclosed yet

**(iv) Name**: “Lead candidate” **Company (Country):** Sanofi/GSK collaboration (France/UK)

**Antigen**: Recombinant trimeric SARS-CoV-2 Spike protein (S)

**Details of platform**: Sanofi's recombinant DNA technology and their Sf9 insect cell expression system

**Route/mode of administration**: presumably i.m. injection

**Safety of Platform**: the combination of a protein-based antigen together with an adjuvant, is well-established and used in a number of vaccines available today

**Advantages**: fast design and relatively rapid production possible

**Clinical Trial Registration**: not yet registered **Start date**: July-September 2020 projected

**Official Name of Trial**: not yet named

**Study design**: Phase I not started yet; **Dosing Regimen**: not known; **Endpoints**: not disclosed yet

**Funding Partners:** BARDA

**Manufacturing partners:** both Sanofi and GSK are global leaders in vaccine development and they have the capacity to generate millions of doses

**Next stage**: Get clinical trials underway and complete the development required for availability of a vaccine by the second half of 2021

**Timeline**: not disclosed yet

Viral Vector-Based (Nonreplicating)

(i) **Name**: AZD1222 (formerly ChAdOx1 nCoV-19) **Company (Country):** University of Oxford/Astra Zeneca collaboration (UK)

**Antigen**: SARS-CoV-2 Spike glycoprotein (S)

**Details of platform**: AZD1222 derives from a chimpanzee viral vector (ChAdOx1), which is a weakened version of a common cold adenovirus with the Spike-encoding region cloned into the E1 locus. Although it infects this primate, it is genetically altered so that it is incapable of viral spread in humans. The same vector modality is in vaccine candidates for influenza, tuberculosis, Chikungunya and Zika viruses.

**Route/mode of administration**: i.m. injection

**Safety of Platform**: Vaccines made from the ChAdOx1 viral vector platform for over 10 different pathogens have been tested in thousands of volunteers (1 week to 90 y.o.) to date and are safe and well tolerated, although they can cause temporary side effects, such as a temperature elevation, headache or sore arm

**Advantages**: favorable safety and tolerability profile of the platform

**Clinical Trial Registration**: NCT04324606 (NCT04400838; PhII/III) **Start date**: April 23, 2020

**Official Name of Trial**: A Phase I/II Study to Determine Efficacy, Safety and Immunogenicity of the Candidate Coronavirus Disease (COVID-19) Vaccine ChAdOx1 nCoV-19 in UK Healthy Adult Volunteers

**Study design**: Phase I/II single-blinded, randomized multicenter participants recruited in UK; n=1102 volunteers 18–55 y.o. good health, n=551 in active arm ChAdOx1 nCoV-19 and n=551 in control arm using MenACWY vaccine (same vector but against meningitis); **Dosing Regimen**: Single 5 × 10^10^ vp (a few dual dose with boost of 2.5 × 10^10^ vp) **Endpoints**: Primary - Number of virologically confirmed (PCR positive) symptomatic cases of COVID-19; Occurrence of serious adverse events (SAEs) throughout the study duration; Secondary - safety/tolerability/reactogenicity/immunogenicity

**Partners:** Vaccitech (role taken over by Astra Zeneca); Operation Warp Speed

**Funding Partners:** CEPI, GAVI, BARDA

**Manufacturing partners:** Serum Institute of India and WuXi Biologics Inc., Vaccines Manufacturing and Innovation Centre (Pall Life Sciences, a unit of Danaher Corp.; Cobra Biologics AB; Dutch CDMO Halix B.V.; Merck; and Oxford Biomedica plc, Advent Srl)

**Next stage**: Preliminary human data June 2020; Phase III efficacy of 5000 volunteers by Fall 2020

**Timeline**: 20-40 million doses of AZD1222 by September 2020 and 400 million by end 2020

(ii) **Name**: Ad5-nCoV **Company (Country):** CanSino Biologics Inc. (China)

**Antigen**: Full-length SARS-CoV-2 Spike glycoprotein (S)

**Details of platform**: Ad5 vectors are well studied and can be grown into high titer stable stocks, they infect non-dividing and dividing cells, they are maintained in cells as an episome; the essential E1A and E1B genes are deleted and replaced by an expression cassette with a high activity cytomegalovirus immediate early (CMV) promoter, which drives expression of the target S protein

**Route/mode of administration**: i.m. injection into deltoid

**Safety of Platform**: In general, safe and well tolerated; however, can be dangerous in immunocompromised individuals. One drawback is that there could be pre-existing neutralizing Abs to the Ad5 vector in some adults

**Advantages**: well-tested vector in gene therapy and vaccination trials (MERS and Ebola) but could be difficult for large-scale manufacturing

**Clinical Trial Registration**: (i) NCT04313127; (ii) ChiCTR2000031781/(NCT04341389) **Start date**: (i) March 16, 2020; (ii) April 12, 2020

**Official Name of Trial**: (i) A Single-center, Open-label Dose-escalating Phase I Clinical Trial to Evaluate Recombinant Novel Coronavirus Vaccine (Adenovirus Type 5 Vector) in Healthy Adults Aged 18-60 Years Old (ii) A randomized, double-blinded, placebo-controlled phase II clinical trial for Recombinant Novel Coronavirus (2019-nCOV) Vaccine (Adenovirus Vector) in healthy adults aged above 18 years

**Study design**: (i) Phase I, non-randomized open label dose-escalating with three cohorts (low-, middle, high-dose for 18-60 y.o, n=108; (ii) Phase II randomized, double-blinded, placebo-controlled with 3 groups (low- and middle-dose, placebo) n=500; **Dosing Regimen**: (i) Single dose (5 x 10^10^, 10^11^, 1.5 x 10^11^ vp Ad5-nCoV n=36/cohort (ii) single dose (5 x 10^10^ vp, n=125; 10^11^ vp, n=250; placebo n=125); **Endpoints**: Primary – General safety including any adverse events (AEs) up to 7 (or 14 for Phase II) days after injection; Secondary – Safety up to 6 months and various immunogenicity indices up to 6 months for both Phase I and II trials

**Partners:** vaccine co-developed with Beijing Institute of Biotechnology; Academy of Military Medical Sciences. PLA of China Jiangsu Province Centers for Disease Control and Prevention, Hubei Provincial Center for Disease Control and Prevention, Tongji Hospital);(ii) Jiangsu Provincial Center for Disease Control and Prevention; Canadian Immunization Research Network at the Canadian Center for Vaccinology; (iii) Canadian Center for Vaccinology (NCT04398147)

Funding Partners: CanSino

**Manufacturing partners:** CanSino, others (not known)

**Next stage**: evaluate Phase II trial data and scale-up production

**Timeline**: preliminary data from Phase I just published (see below) but no exact timelines on production and scale-up reported

(iii) **Name**: Ad26 SARS-CoV-2 (+ 2 back ups) **Company (Country):** Johnson and Johnson (USA)

**Antigen**: undisclosed

**Details of platform**: Janssen's AdVac^®^ and PER.C6^®^ technologies allow for over a million doses to be produced from a 1000 liter bioreactor since the cells grow to high density in suspension culture; the non-replicating Ad26 vector (E1/E3 genes deleted) is likely better than Ad5 since less likelihood of preexisting antibodies to the vector

**Route/mode of administration**: i.m. injection into deltoid

**Safety of Platform**: their particular platform technology is used for an investigational Ebola vaccine in Africa and has also been used for their Zika, RSV and HIV vaccine candidates and appears to be safe

**Advantages**: well-tested vector in gene therapy and vaccination trials with transport/storage at 2-8 °C for 6 months

**Clinical Trial Registration**: not yet registered **Start date**: September 2020 projected

**Official Name of Trial**: not known yet

**Partners:** Janssen Pharmaceutical Companies of Johnson & Johnson, Beth Israel Deaconess Medical Center; Operation Warp Speed

**Funding Partners:** BARDA cofunding $1 billion^+^

**Manufacturing partners:** Emergent BioSolutions Inc., Catalent

**Next stage**: Quick rollout of Phase I in September and EUA by early 2021

**Timeline**: goal of providing global supply of more than one billion doses of a vaccine (unspecified timing)

Viral Vector-Based (Replicating)

(i) **Name**: “Lead candidate” **Company (Country):** Institut Pasteur (France)/Merck (USA)

**Antigen**: undisclosed (“selected, incorporated protein antigens from SARS-CoV-2”)

**Details of platform**: measles vaccine vector exclusively licensed to Themis (purchased by Merck) from Institut Pasteur, with the capacity to insert multiple large recombinant protein antigens includes auto-adjuvant effect by live attenuated replicating vector

**Route/mode of administration**: i.m. injection into deltoid presumably

**Safety of Platform**: the technology that Themis uses to develop its vaccine candidates is based on the measles vaccine, which has been used for decades to immunize safely millions around the world; their approach was used to develop a vaccine candidate against SARS, and CEPI has previously partnered with Themis and Institut Pasteur to harness this technology to develop vaccine candidates against Chikungunya, MERS, and Lassa fever

**Advantages**: proven advanced development and manufacturing capabilities for this platform

**Clinical Trial Registration**: not yet registered **Start date**: September 2020 projected

**Official Name of Trial**: still in preclinical testing phase

**Partners:** University of Pittsburgh

**Funding Partners:** CEPI $4.9 M

**Manufacturing partners:** Merck**/**Themis/ABL Europe

**Next stage**: rollout of Phase I this year

**Timeline**: unspecified timing

Inactivated Virus

(i) **Name**: CoronaVac (formerly PiCoVacc) **Company (Country):** Sinovac Biotech (China)

**Antigen**: whole virus, with initial reports that the RBD within the Spike protein is the main immunogen (Gao et al., 2020)

**Details of platform**: the inactivated viral vaccine platform is straightforward and used extensively; here, the CN-2 SARS-CoV-2 virus was plaque-purified and passaged several times in Vero cells prior to inactivation with β-propiolactone, verification of inactivation, followed by stringent purification protocols and mixing with an alum adjuvant (Gao et al., 2020)

**Route/mode of administration**: i.m. injection

**Safety of Platform**: vaccines made from inactivated virus are used throughout the world with a generally excellent safety profile

**Advantages**: straightforward process; favorable safety and tolerability profile

**Clinical Trial Registration**: NCT04352608; (NCT04383574) **Start date**: April 16, 2020

**Official Name of Trial**: A Randomized, Double-Blinded, Placebo-Controlled, Phase I/II Clinical Trial, to Evaluate the Safety and Immunogenicity of the SARS-CoV-2 Inactivated Vaccine in Healthy Adults Aged 18-59 Years

**Study design**: Phase I/II, randomized double-blinded, placebo-controlled with various groups of 18–59 y.o. volunteers, n=744 (n=144 for Phase I and n=600 for Phase II); **Dosing Regimen**: Dual dosing at Day 0 and Day 14 or 28 of medium dose or high dose or placebo in 0.5 ml; **Endpoints**: Primary – General safety including adverse events (AEs) to 28 days and immunogenicity indices compared between day 0 and day 28; Secondary: Safety and various immunogenicity indices up to Day 56

**Partners:** Sinovac Research and Development Co., Ltd.

**Funding Partners:** Advantech Capital and Vivo Capital ($15M)

**Manufacturing partners:** partnering with Dynavax Technologies Corp for use of their adjuvant CpG 1018, which is currently used in an FDA-approved Hepatitis B Vaccine (Recombinant)

**Next stage**: starting Phase III by end of 2020

**Timeline**: 100 million doses/year projected

(ii) **Name**: COVID-19 vaccine **Company (Country):** Sinopharm (China)

**Antigen**: whole virus

**Details of platform**: The inactivated viral vaccine platform is straightforward and used extensively; no details on this particular vaccine could be found

**Route/mode of administration**: i.m. injection

**Safety of Platform**: vaccines made from inactivated virus are used throughout the world with a generally excellent safety profile

**Advantages**: straightforward process; favorable safety and tolerability profile

**Clinical Trial Registration**: ChiCTR2000031809; ChiCTR2000032459* **Start date**: April 11, 2020

**Official Name of Trial**: A randomized, double-blind, placebo parallel-controlled phase I/II clinical trial for inactivated Novel Coronavirus Pneumonia vaccine (Vero cells)

**Study design**: Phase I/II randomized, double-blinded, placebo-controlled conducted in Jiaozuo, Henan Province on healthy 6 y.o. and up; 96 volunteers injected by April 23; with n=216 to be injected with either low-, mid-, or hi-doses or placebo n=72 in Phase I; multiple groups in Phase II n=876 to be injected with either low-, mid-, or hi-doses or placebo n=292; **Endpoints**: Primary – adverse events; Secondary – Multiple safety and immunogenicity parameters up to 1 year post-injection

**Partners:** Wuhan Institute of Biological Products under the China National Pharmaceutical Group, Sinopharm, and the Wuhan Institute of Virology (WIV)/Beijing Institute of Biological Products*

**Funding Partners:** not known

**Manufacturing partners:** unknown

**Next stage**: unknown

**Timeline**: unknown

*Two trials are registered in China with different clinical research groups but under “Sinopharm” and it is unclear if they are using the same vaccine candidate*

Live Attenuated Virus/Other

(i) **Name**: “Lead” **Organization (Country):** University of Hong Kong (China)

**Antigen**: undisclosed which SARS-CoV-2 protein(s) used

**Details of platform**: a weakened version of the flu virus adapted to express surface protein of the COVID-19 virus

**Route/mode of administration**: intranasal

**Safety of Platform**: approach has been used previously to develop preclinical vaccine candidates against MERS

**Advantages**: straightforward process used for several licensed human vaccines; existing infrastructure can be used

**Clinical Trial Registration**: not yet registered **Start date**: July 2020 projected

**Official Name of Trial**: still in preclinical phase

**Funding Partners:** CEPI

**Manufacturing partners:** dependent on mainland China for manufacturing, no specific partner mentioned

**Next stage**: getting Phase I trials underway by summer 2020

**Timeline**: unknown

### Data From First Clinical Trials and NHPs for COVID-19 Vaccines in Development

#### mRNA-1273 Moderna Study

In a May 18 press release of interim data^15^ and ensuing evaluation (Callaway, 2020c), the company reported that in the initial eight participants mRNA-1273 on the two lower doses (25 and 100 µg) evoked neutralizing antibody titer levels reaching or exceeding neutralizing antibody titers generally seen in convalescent sera as measured by a plaque reduction neutralization assay against live SARS-CoV-2. mRNA-1273 was generally safe and well tolerated and provided full protection against viral replication in the lungs in a mouse challenge model. In view of these data, the company is forging on with Phase II and III studies with an anticipated dose for Phase III using between 25 µg and 100 µg RNA. Since the level of neutralizing antibodies against SARS-CoV-2 is generally quite variable, and in some cases undetectable, in people who have recovered from COVID-19 without hospitalization (Wu et al., 2020b), it remains to be seen how effective the Moderna vaccine candidate will be at providing long-term immunity. This should come into focus in the upcoming large clinical trials.

#### AZD1222 (ChAdOx1 nCoV-19) University of Oxford Study

In this preclinical trial (van Doremalen et al., 2020), NHPs (rhesus macaques) were administered a very high dose of SARS-CoV-2 after receiving a single dose of the vaccine (very similar to regimen being used in the current clinical trials). The active treatment group of animals (n=6) produced elevated levels of SARS-CoV-2 neutralizing antibodies compared to no increase in the control group (n=3). There were indistinguishable amounts of virus in the nasal compartment compared to control animals but significantly reduced viral load in the lungs. The vaccinated NHPs were free of clinical-grade pneumonia, in contrast to the controls. Although the levels of antibodies and cell-mediated cytokine responses in the vaccinated NHPs were substantial, nobody knows yet whether this level of immune response will be protective in humans and how long that protection will last.

#### Ad5-nCoV CanSino Study

In this complete analysis of Phase I data (Zhu F. et al., 2020) obtained between March 16 and March 27, 2020, n=108 participants (51% male, 49% female; mean age 36 y.o.) with equal numbers receiving low-, mid- and high-doses of the vaccine, there were mild-moderate AEs in 75%–83% in each group (injection site pain, fever, fatigue, headache, and muscle pain) with no SAEs within the first 28 days post-vaccination. Neutralizing antibodies increased significantly in the 14–28 day timeframe post-vaccination, along with specific T-cell responses. The data indicate that this Ad5-vectored COVID-19 vaccine warrants further investigation and they will continue with their Phase II randomized, double-blinded, placebo-controlled study (see above).

#### CoronaVac (PiCoVacc) Sinovac Biotech Study

In this preclinical NHP study (Gao et al., 2020) there was robust SARS-CoV-2-specific neutralizing antibody responses in the rhesus macaques that received three doses of the inactivated virus, which importantly afforded partial to full protection from clinical signs of lung injury after viral challenge. In addition, there were no signs of ADE.

## Concluding Remarks

The world is anxiously awaiting a safe, effective vaccine to protect against COVID-19 in order to resume a “normal” lifestyle, free from public health agency/government lockdowns and fear of ongoing pandemic waves over the coming months-years. Never before in the modern era of science has the accumulation of scientific papers in preprint archives (BioRxiv/MedRxiv, ≈5000 preprints) and peer-reviewed publications (PubMed, ≈22,000 papers) reached such exponential heights in such a short period with thousands of “all-things-considered” COVID topics. Mobilization of data sharing and joint global efforts toward this vaccine goal are monumental. It is hoped that the collaborative framework (such as ACTIV, and others around the world) for prioritizing vaccine and drug candidates, streamlining clinical trials, coordinating regulatory processes and/or leveraging assets among various partners to rapidly respond to the COVID-19 and future pandemics will soon be a reality. However, a successful path forward will be challenging and is certainly not guaranteed. For instance, no efficacious and approved vaccine for HIV/AIDS has come forward in over 30 years. The main HIV surface protein for host cell entry is covered in sugars, as is the Spike protein of SARS-CoV-2, but to a lesser extent (Watanabe et al., 2020). Will this site-specific glycan shield provide difficulties in target antigen recognition, if not properly reconstituted, in some of the vaccine platforms? Will it be necessary to target more than just the Spike protein, which most approaches are banking on? Cryptic epitopes for antibody recognition need to be considered (Yuan et al., 2020) and multivalent formulations may be required to generate effective long-lasting immunity with the ideal target product profile (**Table 1**). SARS-CoV-2 antibody-based therapeutics derived from consortia such as CoVIC^16^ (Coronavirus Immunotherapy Consortium) and other research groups/biopharma for COVID-19 have already entered clinical trials^17^. These biologics, as well as repurposed drugs, will likely provide mid-term solutions while we wait patiently for the much-anticipated vaccine.

## Author Contributions

CF and AA conceived the design and concepts. CF wrote the manuscript. CL contributed key information for **Table 1**. All authors contributed to the article and approved the submitted version.

## Funding

This research was funded by Queen's University Special Research Project 379415 (CDF).

## Conflict of Interest

AA is the founder and managing director of Novateur Ventures, Inc. CL is a consultant with Novateur Ventures Inc. CF is a scientific advisor for Novateur Ventures Inc.

CF declares that the research was conducted in the absence of any commercial or financial relationships that could be construed as a potential conflict of interest.

## Footnotes

1 https://coronavirus.jhu.edu/map.html [accessed June 7, 2020].

2 https://www.nih.gov/news-events/news-releases/nih-clinical-trial-shows-remdesivir-accelerates-recovery-advanced-covid-19 [accessed May 23, 2020].

3 https://www.clinicaltrialsarena.com/news/french-early-trial-tocilizumab-covid-19/ [accessed May 23, 2020].

4 https://www.historyofvaccines.org/timeline/polio [accessed May 24, 2020].

5 https://www.ema.europa.eu/en/documents/leaflet/ebola-vaccine-development-2014-2019_en.pdf [accessed May 23, 2020].

6 https://www.who.int/emergencies/diseases/novel-coronavirus-2019/global-research-on-novel-coronavirus-2019-ncov [accessed May 26, 2020].

7 https://cepi.net/covid-19/ [accessed May 26, 2020].

8 https://milkeninstitute.org/covid-19-tracker [accessed May 26, 2020].

9 https://www.biocentury.com/coronavirus [accessed May 26, 2020].

10 https://www.medicalcountermeasures.gov/app/barda/coronavirus/COVID19.aspx [accessed May 26, 2020].

11 https://www.gavi.org/ [accessed May 26, 2020].

12 https://www.nih.gov/research-training/medical-research-initiatives/activ [accessed May 26, 2020].

13 https://www.cnn.com/2020/04/29/politics/operation-warp-speed-coronavirus-vaccine/index.html [accessed May 26, 2020].

14 https://www.npr.org/sections/coronavirus-live-updates/2020/05/15/857014274/trump-touts-operation-warp-speed-coronavirus-vaccine-effort [accessed May 24, 2020].

15 https://investors.modernatx.com/news-releases/news-release-details/moderna-announces-positive-interim-phase-1-data-its-mrna-vaccine [accessed May 25, 2020].

16 https://covic.lji.org/

17 https://www.fiercebiotech.com/biotech/lilly-starts-first-clinical-trial-covid-19-antibody-therapy

## Abbreviations

COVID-19, Coronavirus disease 2019; SARS, Severe Acute Respiratory Syndrome; SARS-CoV-1, Severe acute respiratory syndrome coronavirus 1; SARS-CoV-2, Severe acute respiratory syndrome coronavirus 2; MERS, Middle East Respiratory Syndrome; S, SARS-CoV-2 Spike protein; E, SARS-CoV-2 Envelope protein; M, SARS-CoV-2 Membrane protein; N, SARS-CoV-2 Nucleocapsid protein; RBD, receptor-binding domain; TMPRSS-2, transmembrane protease, serine 2; Ab, antibody; Ad, adenovirus; ACE2, angiotensin converting enzyme 2; CMV, cytomegalovirus; RSV, respiratory syncytial virus; CEPI, Coalition for Epidemic Preparedness Innovations; GAVI, GAVI The Vaccine Alliance (formerly Global Alliance for Vaccines and Immunization); BARDA, Biomedical Advanced Research and Development Authority; WHO, World Health Organization; EMA, European Medicines Agency; FDA, Food and Drug Administration; NIH, National Institutes of Health; NIAID, National Institute of Allergy and Infectious Diseases; EUA, emergency use authorization; ACTIV, Accelerating COVID-19 Therapeutic Interventions and Vaccines; EU, European Union; CRP, C-reactive protein; MIS-C, multisystem inflammatory syndrome in children; ORF, open-reading frame; VAERD, vaccine-associated enhanced respiratory disease; ADE, antibody-dependent enhancement; ADCC, antibody-dependent cellular cytotoxicity; CDCC, complement-dependent cellular cytotoxicity; IL-6, interleukin 6; IFN, interferon; LNP, lipid nanoparticle; VLP, virus-like particle; PCR, polymerase chain reaction; EP, electroporation; MenACWY, meningococcal vaccine that protects against four strains of the bacteria – A, C, W and Y; NHP, non-human primate; AE, adverse event; SAE, serious adverse event; HCW, health-care worker; COGS, cost of goods sold; i.m., intramuscular; i.d., intradermal; vp, viral particles; y.o., years old.
